# Detection of Tumor-Associated Membrane Receptors on Extracellular Vesicles from Non-Small Cell Lung Cancer Patients via Immuno-PCR

**DOI:** 10.3390/cancers13040922

**Published:** 2021-02-22

**Authors:** Christiane Stiller, Kristina Viktorsson, Elizabeth Paz Gomero, Petra Hååg, Vasiliki Arapi, Vitaliy O. Kaminskyy, Caroline Kamali, Luigi De Petris, Simon Ekman, Rolf Lewensohn, Amelie Eriksson Karlström

**Affiliations:** 1 Department of Protein Science, School of Engineering Sciences in Chemistry, Biotechnology and Health, KTH Royal Institute of Technology, AlbaNova University Center, SE-10691 Stockholm, Sweden; cstiller@kth.se (C.S.); epaz@kth.se (E.P.G.); 2 Biomedical Centre, Department of Pharmaceutical Biosciences, Uppsala University, SE-75123 Uppsala, Sweden; 3 Department of Oncology-Pathology, Karolinska Institutet, SE-17177 Stockholm, Sweden; kristina.viktorsson@ki.se (K.V.); petra.haag@ki.se (P.H.); varapi@caredx.com (V.A.); Vitaly.Kaminsky@ki.se (V.O.K.); caroline.kamali@ki.se (C.K.); luigi.depetris@ki.se (L.D.P.); simon.ekman@ki.se (S.E.); rolf.lewensohn@ki.se (R.L.); 4 Theme Cancer, Medical Unit Head and Neck, Lung, and Skin Tumors, Thoracic Oncology Center, Karolinska University Hospital, SE-17177 Stockholm, Sweden

**Keywords:** immuno-PCR, small extracellular vesicles, liquid biopsies, non-small-cell lung cancer, EGFR, tyrosine kinase inhibitors, longitudinal treatment monitoring, biomarkers, affibody, protein–DNA conjugation

## Abstract

**Simple Summary:**

Lung cancer is often detected at late stages when metastases are present and the genomic make-ups of the tumors are heterogeneous. Analyses of genomic alterations in non-small-cell lung cancer (NSCLC) have revealed mutated tumor-associated membrane receptors and fusion proteins, which can be targeted via tyrosine kinase inhibitors (TKIs). TKIs initially often have a good effect, but a fraction of the tumor lesions may develop resistance through additional mutations in the targeted kinases or by increased expression/function of other membrane receptors. Detection of TKI-bypassing mechanisms is difficult in tissue biopsies as these analyze only a subpart of tumors or lesions. Liquid biopsies based on tumor-secreted small extracellular vesicles (sEVs) into body fluids can assess tumor heterogeneity. We present an immuno-PCR method for the detection of the epidermal growth factor receptor (EGFR), the human epidermal growth factor receptor 2 (HER2), and the insulin-like growth factor 1 receptor (IGF-1R) on sEVs. Initial investigations of sEVs from *EGFR*-mutant NSCLC tumor cells or pleural effusion (PE) fluid from patients with NSCLC or benign diseases showed different protein profiles for individual sEV samples. Further development of the immuno-PCR could complement DNA/mRNA-based assays detecting kinase mutations to allow longitudinal treatment monitoring of diverse TKI-bypassing mechanisms.

**Abstract:**

Precision cancer medicine for non-small-cell lung cancer (NSCLC) has increased patient survival. Nevertheless, targeted agents towards tumor-associated membrane receptors only result in partial remission for a limited time, calling for approaches which allow longitudinal treatment monitoring. Rebiopsy of tumors in the lung is challenging, and metastatic lesions may have heterogeneous signaling. One way ahead is to use liquid biopsies such as circulating tumor DNA or small extracellular vesicles (sEVs) secreted by the tumor into blood or other body fluids. Herein, an immuno-PCR-based detection of the tumor-associated membrane receptors EGFR, HER2, and IGF-1R on CD9-positive sEVs from NSCLC cells and pleural effusion fluid (PE) of NSCLC patients is developed utilizing DNA conjugates of antibody mimetics and affibodies, as detection agents. Results on sEVs purified from culture media of NSCLC cells treated with anti-EGFR siRNA, showed that the reduction of EGFR expression can be detected via immuno-PCR. Protein profiling of sEVs from NSCLC patient PE samples revealed the capacity to monitor EGFR, HER2, and IGF-1R with the immuno-PCR method. We detected a significantly higher EGFR level in sEVs derived from a PE sample of a patient with an *EGFR*-driven NSCLC adenocarcinoma than in sEVs from PE samples of non-*EGFR* driven adenocarcinoma patients or in samples from patients with benign lung disease. In summary, we have developed a diagnostic method for sEVs in liquid biopsies of cancer patients which may be used for longitudinal treatment monitoring to detect emerging bypassing resistance mechanisms in a noninvasive way.

## 1. Introduction

Even though tissue biopsies are a standard diagnostic method for tumor malignancies, they have a limited ability to detect the signaling heterogeneity of tumors, as one only analyzes a subpart of the tumor. In particular, this is an obstacle in analyses of metastatic tumors where oncogenic drivers may be diverse in different lesions of the patient. Liquid biopsies are therefore increasingly recognized for their ability to detect and monitor tumors in a less invasive way [1,2,3]. Liquid biopsy methods also hold promise for longitudinal monitoring of tumor responses to a given treatment due to their fast repeatability and low cost per patient. Certain liquid biopsies for circulating tumor cells (CTCs) or circulating tumor DNA (ctDNA) are already available as clinical diagnostics approved by the U.S. Food and Drug Administration (FDA) [1,4,5]. Approaches based on CTCs are to some extent hampered as a consequence of their low number in patient blood samples and due to the lack of straightforward isolation or detection methods. ctDNA on the other hand is more abundant in body fluids of cancer patients but requires targeted analytical approaches where the tumor mutations to be monitored are limited in numbers or already known from the tumor tissue [6,7]. Moreover, ctDNA may not reveal all signaling events going on in a tumor, which also take place on miRNA-, mRNA-, protein expression and signaling levels. Therefore, it is unlikely that analyses of CTCs or ctDNA alone will allow detection of all tumor malignancies and/or their treatment response or resistance. An additional biomarker source is small extracellular vesicles (sEVs) or exosomes generated via the endosomal system, which are membrane-encapsulated vesicles containing a set of proteins, nucleic acids, lipids, and metabolites resembling in part their parental cells [3]. sEVs are secreted from all cell types and present in all body fluids. Because sEVs are higher in number and with diameters below 200 nm smaller in size than CTCs, they are easier to separate from blood cells, and their membrane protects their cargo, allowing for reproducible analysis after storage [8,9].

Liquid biopsies are especially interesting for longitudinal treatment monitoring of lung-cancer (LC) patients as rebiopsy of tumor lesions localized to the lung is challenging and not always successful [1]. LC is one of the most common tumor types, and patients are often diagnosed when the tumor has reached an advanced stage. Characterization of aberrant oncogenic signaling in non-small-cell lung cancer (NSCLC) has resulted in precision cancer medicine treatment approaches via tyrosine kinase inhibitors (TKIs) towards mutated *epidermal growth-factor receptor (EGFR)* or *EML4-ALK* fusions [10,11,12,13]. These mutations or fusions cause constitutive kinase signaling which enables proliferation of the NSCLC tumor [11,12,13,14]. Patients treated with first generation TKIs do usually not experience a complete response and resistance is frequently acquired [11,12,13,14], which may be a consequence of compensatory mutations in the *EGFR* or *ALK* gene. Such mutations can be combatted by 2nd or 3rd generation TKIs [13,15,16]. However, EGFR- or ALK-TKI resistance may also be caused through bypassing mechanisms including amplification or activation of other transmembrane receptors or their downstream signaling, e.g., MET, HER2/ERBB2, AXL, IGF-1R, FGFR1, EPHA2, RAS/RAF/MAPK, PI3K/AKT, mTOR, and NF-κBs for EGFR-TKI [11,14,17,18], as well as EGFR and HER2 in relation to ALK-TKI resistance [13].

**Figure 1:**
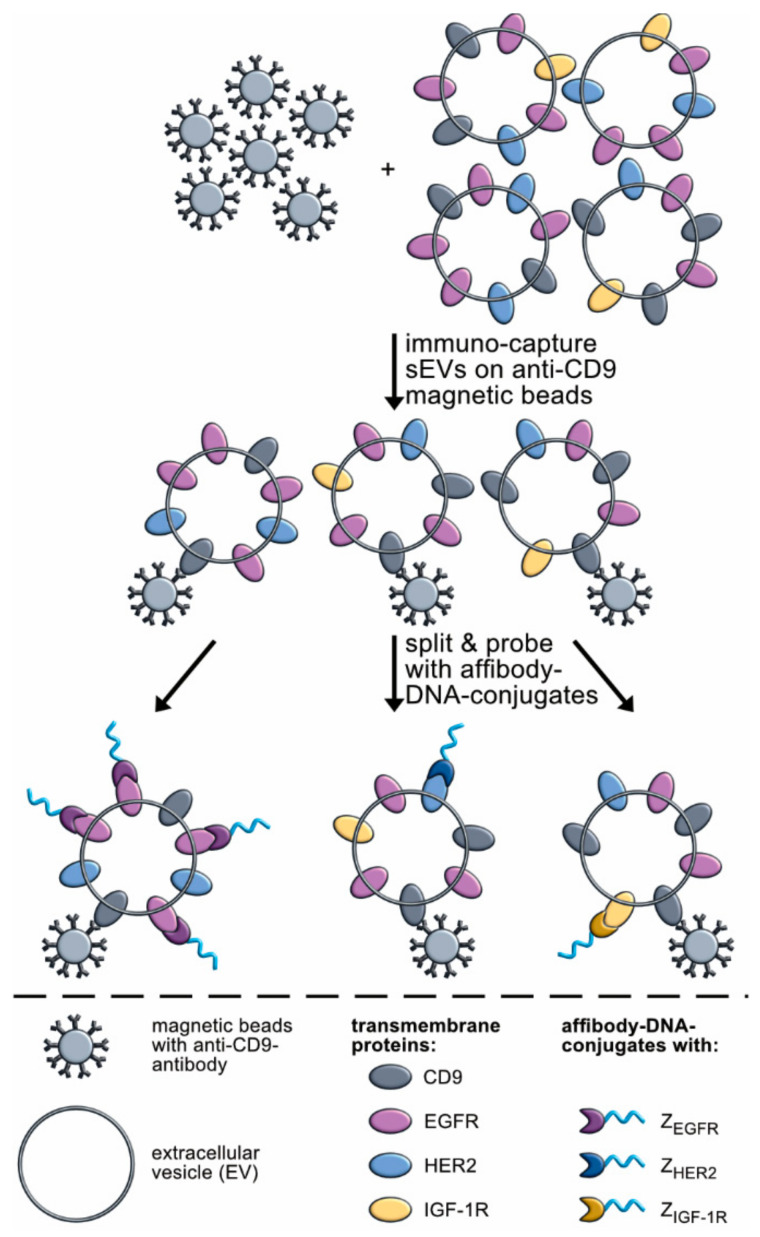
Overview of the developed immuno-PCR assay. Small extracellular vesicles (sEVs) (grey) carrying the transmembrane receptors EGFR, HER2, and IGF-1R (light purple, blue and yellow) were immuno-captured on CD9 antibody-coated magnetic beads (grey with black) before division into subsamples and addition of individual affibody–DNA conjugates (dark-purple, blue, and yellow proteins with light-blue DNA). Afterwards, bound conjugates were amplified and analyzed via RT-PCR.

While the monitoring of *EGFR* mutations via ctDNA in plasma combined with exosomal RNA (exoRNA) has already been shown [19,20], the detection of bypass resistance mechanisms driven by altered protein signaling is not as straightforward. Here, analysis of the protein cargo on or in sEVs could potentially allow the detection of resistant tumor parts during treatment early on [1,2,3]. Indeed, methods like antibody arrays [21], fluorescence-activated cell sorting (FACS) surface profiling [22], and proteomic profiling via mass spectrometry (MS) [23] have already been employed for the analysis of NSCLC-derived sEVs. However, some of these analyses are not easily transferred into clinical applicable methods as they require specialized equipment and trained personnel or are time-consuming and expensive [24,25]. In addition, some studies lack information on the assay reproducibility making it difficult to judge if longitudinal application is feasible. For longitudinal studies, it is crucial to have a robust measurement system, which can deliver stable results on the same sample over weeks/months as only this will allow to track changes in the protein profile of sEVs from individual patients. Moreover, methods need to be easy to implement in clinical laboratories. As enzyme-linked immunosorbent assays (ELISAs) and real time polymerase chain reaction (RT-PCR) instruments are broadly used, we aimed to develop a diagnostic tool for sEVs in liquid biopsies of tumor patients based on immuno-PCR towards growth-factor receptors with oncogenic function (Figure 1). For the immuno-PCR’s affinity part protein–DNA conjugates are needed, and we decided to use affibody–DNA conjugates. Affibodies are small antibody-mimetics based on the Z-domain derived from protein A, and several versions have been generated by selection against different targets using diverse display techniques from libraries constructed by randomizing 13 of their 58 amino acids. Well-studied variants binding to several cancer-related targets exist [26]. As we recently developed a method for conjugation of the Z-domain with DNA [27], we anticipated that affibody–DNA conjugates could be obtainable in an analogue fashion. For this study we used the four affibodies Z_EGFR_, Z_HER2_, Z_HER3_, and Z_IGF-1R_, which show nano- to pico-molar affinities against their respective targets, the extracellular domains of transmembrane receptors epidermal growth factor receptor (EGFR), human epidermal growth factor receptor 2 (HER2), human epidermal growth factor receptor 3 (HER3), and insulin-like growth factor 1 receptor (IGF-1R), while also having negligible binding toward related receptors or highly abundant body proteins (Table S1) [28,29,30,31,32,33]. Therefore, these affibodies have successfully been used as tracers in PET imaging of xenografted tumors in mice and/or in patients with different tumor types [31,32,33,34]. We show that these affibody Z*_x_*–DNA conjugates are useful reagents in monitoring sEVs from cultivated NSCLC cells. Moreover, we reveal that our immuno-PCR differentiates sEVs from liquid biopsies, i.e., malignant pleural effusion (MPE) fluid of patients with EGFR-driven NSCLC, from patients with different NSCLC genotypes as well as from PE fluid from patients with benign disease.

## 2. Results

### 2.1. Z_x_–DNA Conjugates Are Suitable Reagents for Immuno-PCR

To obtain Z*_x_*–DNA conjugates for the study the four affibodies Z_EGFR_, Z_HER2_, Z_HER3_, and Z_IGF-1R_ were produced with a C-terminal Sortase A recognition motif, and protein integrity was confirmed by matrix-assisted laser desorption/ionization time-of-flight (MALDI-TOF) mass spectrometry (Figure S1). Next, 5′-G_3_-modified DNA was coupled to the different affibodies to generate Z_EGFR_–, Z_HER2_–, Z_HER3_– and Z_IGF-1R_–DNA via Sortase A3* catalysis. All conjugates were found to be pure by analytical reversed phase-high performance liquid chromatography (RP-HPLC) (Figure S2). The amplification efficiencies of the four Z*_x_*–DNA conjugates with the nonmodified DNA template in RT-PCR were characterized (Table S2) and found to be 79–84%. The observed different efficiencies in RT-PCR of the Z*_x_*–DNA conjugates hindered an absolute quantification with a common calibration curve. Therefore, the DNA amount of each Z*_x_*–DNA on sEV samples was normalized by dividing it through the background binding of the same Z*_x_*–DNA to beads without sEVs, thereby calculating relative amounts *a_r_*. Such calculation only allows the comparison of identical Z*_x_*–DNA between immuno-RT-PCR samples and not the comparison of different Z*_x_*–DNAs.

### 2.2. Z_x_–DNA Conjugates towards EGFR, IGF-1R, HER2, and HER3 Can Monitor Protein Expression on sEVs from NSCLC Cells

**Figure 2:**
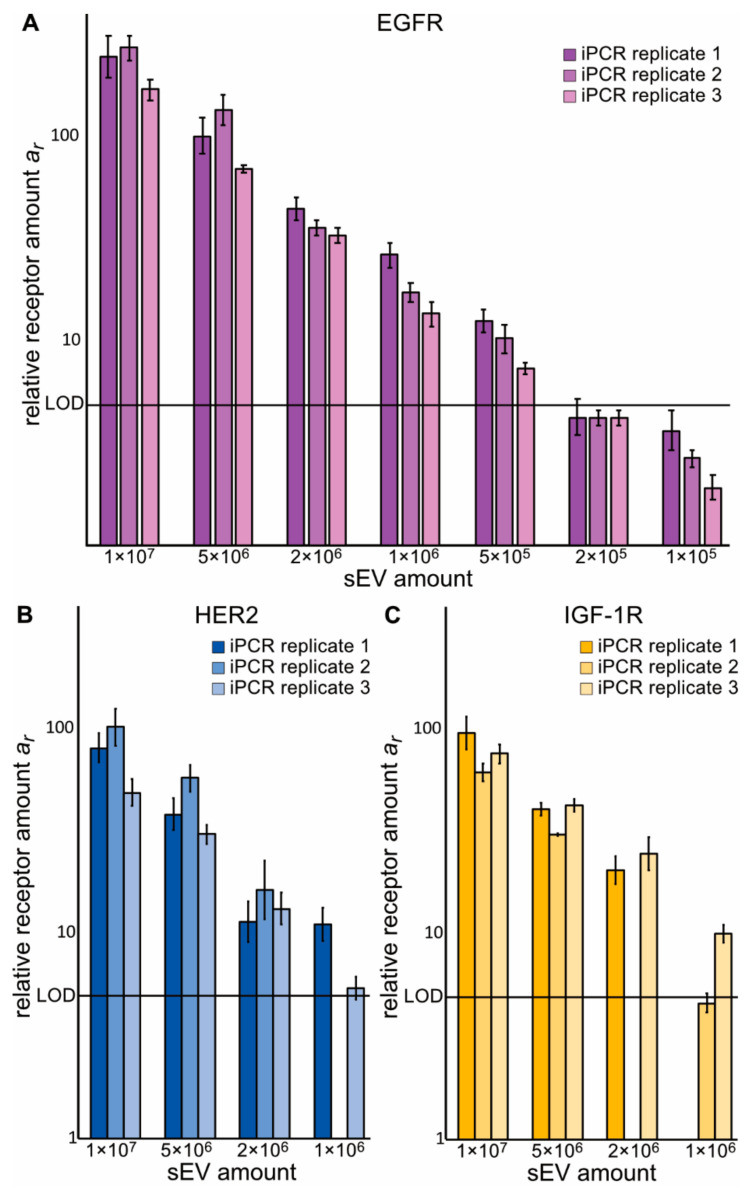
Immuno-PCR analysis of three dilution series of sEVs derived from conditioned cell culture medium of NSCLC H1975 cells. Replicates were performed on the same sEV stock sample isolated from conditioned cell culture medium of NSCLC H1975 cells to investigate the variation of the immuno-PCR method. (**A**): Detection of EGFR with Z_EGFR_–DNA. (**B**): Detection of HER2 with Z_HER2_–DNA. (**C**): Detection of IGF-1R with Z_IGF-1R_–DNA. Each bar represents the result of one immuno-PCR sample, error bar = standard deviation of RT-PCR replicates, LOD = limit of detection.

To test if the Z*_x_*–DNA conjugates could detect their respective targets on sEVs, sEVs purified from conditioned medium of the *EGFR-*mutant NSCLC cell line H1975 were used. sEV preparations were proven to express the ISEV category 1b marker CD9 (Figure S3) [9], while calnexin, an ER-protein previously reported to be absent or under-expressed in sEVs [35], was not detectable in the analyzed sEVs (Figure S3). The Western blot analysis revealed low amounts of EGFR but no HER2 and IGF-1R in the sEV samples, while both EGFR and IGF-1R were clearly expressed in H1975 cells. Particle size and concentrations of sEV samples were measured via nanoparticle tracking analysis (NTA) (Figure S4, top panel). To test the Z*_x_*–DNA conjugates, relative receptor amounts *a_r_* were determined on three dilution series of H1975 cell-derived sEVs in three independent immuno-PCR runs (Figure 2 and Figure S5). Through this analysis, it was possible to detect a difference in expression level of a given receptor between various samples and a clear linear dependency was seen for the dilution series with regard to EGFR, HER2, HER3, and IGF-1R. We determined the lower limit of detection (LLOD) to 3.6 × 10^5^, 5.6 × 10^5^, 19.8 × 10^5^, and 7.8 × 10^5^ H1975 cell-derived sEVs for Z_EGFR_–, Z_HER2_–, Z_HER3_– and Z_IGF-1R_–DNA, respectively (Figure S6). Additionally, we assessed the interassay coefficients of variance (CVs) based on all samples from the dilution series with signals above the methods limit of detection (LOD) to 14, 27, 42 and 22% for Z_EGFR_–, Z_HER2_–, Z_HER3_– and Z_IGF-1R_–DNA, respectively. As the LLOD and the interassay CV for Z_HER3_–DNA were comparably high, this affibody–DNA conjugate was excluded in further experiments.

### 2.3. Immuno-PCR with the Z_EGFR_–DNA Conjugate towards EGFR Can Monitor Alterations in EGFR Expression in sEVs from NSCLC Cells

**Figure 3:**
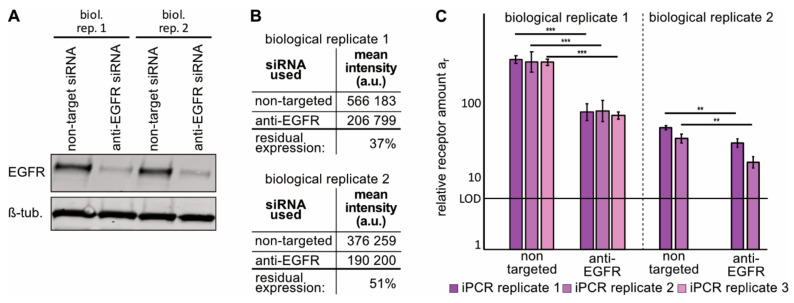
Analysis of EGFR receptor expression on NSCLC H1975 cells and secreted sEVs after anti-EGFR siRNA treatment. The siRNA treatment was performed in two independent biological replicates using EGFR-targeting and nontargeted siRNA in parallel. Efficiency of EGFR knockdown was analyzed by Western blot and FACS on cells before analyzing the corresponding sEVs by immuno-PCR replicates. (**A**): Western blot of total EGFR expression in H1975 cell lysates after siRNA treatment with β-tubulin serving as loading control. (**B**): Summary of FACS quantification of EGFR expression of H1975 cells after EGFR- or non-targeting siRNA treatments (the EGFR FACS data is presented in Figure S7). (**C**): Measured relative receptor amounts *a_r_* on 5 × 10^6^ sEVs isolated from cell-culture media from the EGFR-targeting or nontargeting siRNA-treated H1975 cells. Three or two immuno-PCR replicates for biological replicates 1 and 2 were carried out, respectively. Each bar represents the result of one immuno-PCR sample, error bar = standard deviation of RT-PCR replicates, horizontal line = limit of detection. Significance levels are based on a one-way ANOVA test with subsequent Turkey multicomponent test: ***p < 0.001, **p < 0.01.

As our goal was to develop a method that is able to detect changes in protein patterns on sEV samples during the treatment course of NSCLC patients, we artificially altered the amount of EGFR on the NSCLC cell line H1975, which is driven by mutated EGFR [36]. For this, we treated H1975 cells with either siRNA against EGFR or nontargeting siRNA in two independent biological replicates. Western blot analyses of EGFR expression confirmed reduction in expression after anti-EGFR siRNA treatment on the cells (Figure 3A). Flow cytometry analyses of the total EGFR expression, using an antibody directed towards the cytoplasmatic domain of EGFR, of the same cell samples revealed a residual EGFR expression of about 40 and 50% relative to nontargeted siRNA-transfected H1975 cells for biological replicates 1 and 2, respectively (Figure 3B, Figure S7). Next, sEVs were isolated from the corresponding H1975 conditioned cell-culture media and assayed for their CD9 content to prove sEV identity and the lack of contamination with ER proteins by calnexin detection (Figure S8). The sEVs’ amount and size were measured by NTA (Figure S4, lower four panels). For each biological replicate, independent immuno-PCR replicates were performed using 1.5 × 10^7^ sEVs (Figure 3C and Figure S9). A clear reduction of the relative EGFR amount after anti-EGFR siRNA treatment was visible for the sEV samples (Figure 3C). For the first biological replicate the average residual amount of EGFR on sEVs isolated from cells treated with anti-EGFR siRNA in comparison to nontargeted RNA was measured to be 20 ± 1%, while the second biological replicate showed a reduction to 47 ± 11%. As the FACS-based quantification was performed on whole cells, with an antibody generated against the cytoplasmatic domain of EGFR, thus capturing all EGFR molecules of the cell, whereas sEVs derived from these cells were utilized for the immuno-PCR, detecting the membrane fraction of EGFR only, it is not surprising that the reduction of EGFR expression only matches qualitatively. However, in biological replicate 1, Western blotting analyses of sEVs from EGFR-targeting siRNA treated H1975 cells revealed a reduction to about 20% of EGFR expression relative to sEVs from nontargeted siRNA treated cells (Figure S8). This result agrees quantitatively with the data obtained via the immuno-PCR and shows that this method can capture a decrease in EGFR surface expression on sEVs. Of note, the detected amounts of HER2 and IGF-1R showed only one significant difference between sEVs from H1975 cells treated with EGFR-targeting siRNA vs. nontargeted siRNA in the same samples (Figure S9), further illustrating that the change in EGFR-detection was based on biological differences between the samples.

### 2.4. Immuno-PCR with Z_x_–DNA-Conjugates towards EGFR, HER2 and IGF-1R Can Monitor Protein Expression in sEVs from Liquid Biopsies of NSCLC Patients

**Figure 4:**
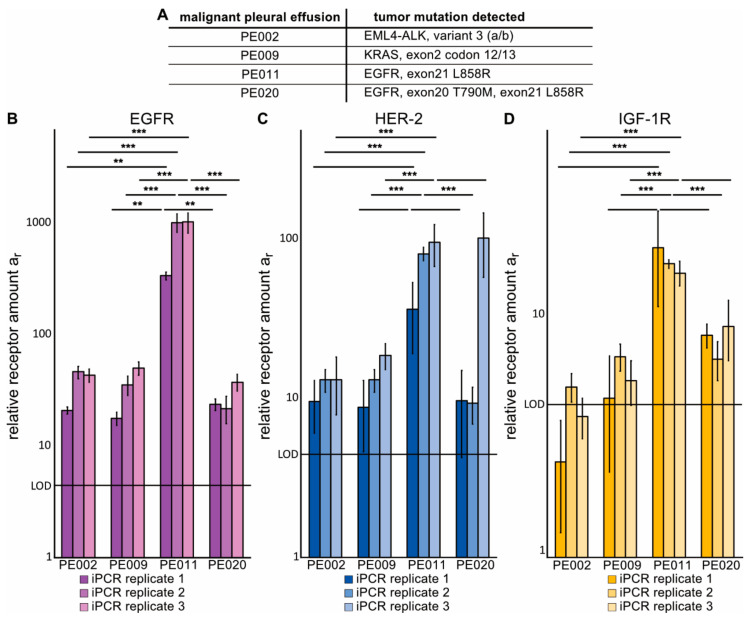
Immuno-PCR analysis of sEVs from malignant pleural effusion (MPE) fluid of advanced NSCLC patients. sEVs were isolated from MPE fluid of four NSCLC patients with adenocarcinoma histology with different clinically validated genomic alterations in tumors and at different time points during their treatment course. Western blot and nanoparticle tracking analysis (NTA) analyses of the sEVs are presented in Figures S10 and S11. (**A**): Summary of patient data matching the samples analyzed in B–D (additional data is presented in Table S3). (**B**–**D**): Relative amounts of EGFR, HER2, and IGF-1R, respectively. Each bar represents the result of one immuno-PCR sample using 6 × 10^7^ sEVs isolated from patient samples, error bar = standard deviation of RT-PCR replicates, LOD = limit of detection. Significance levels are based on a one-way ANOVA test with subsequent Turkey multicomponent test, which was performed independently for the data of each receptor: ***p < 0.001, **p < 0.01, *p < 0.05, no star *p* > 0.05. The third measurement for HER2 on the PE020 sample is an outlier.

After establishing that the immuno-PCR can reveal changes in EGFR expression on NSCLC cells via analysis of sEVs, we investigated if the method can detect the tumor-membrane receptors EGFR, HER2, and IGF-1R in sEVs from NSCLC liquid biopsies. For this purpose, we used malignant pleural effusion (MPE) fluid as a source of sEVs. MPE fluid is seen in about 10 to 15% of all LC-cases at advanced stage and is more common in NSCLC adenocarcinomas likely due to tumor growth in the periphery of the lung [37,38]. MPE draining is primarily done to relieve problems of breathing of the patient, but as MPE fluid is close to the tumor, it also offers a source of tumor material including tumor cells and sEVs as shown by us and others [37,38,39]. In this study, sEVs were isolated from MPE fluid of four NSCLC patients, whose tumors were of adenocarcinoma histology and clinically validated to harbor *EML4-ALK*-fusion (PE002), *KRAS*-mutation (PE009), or mutations in the kinase domain of EGFR (PE011 and PE020) (Figure 4A, Table S3). After quantifying the sEVs concentration by NTA (Figure S10), equal amounts of sEVs were analyzed in three entirely independent immuno-PCR runs (Figure 4B–D) as well as by conventional Western blot (Figure S11). Western blot analysis included detection of CD9 and TSG101 to prove sEVs identity, as well as calnexin to further prove the sEVs [35] as well as to analyze for contamination of ER-proteins. While the Western blot analysis only detected EGFR in sEVs from samples PE009 and PE011, which might stem from different amounts of sEVs loaded, it was possible to detect EGFR by immuno-PCR in sEVs from all four MPE samples with a significantly higher expression level found in PE011. IGF-1R was only detected by Western blotting in PE011 sEVs, which may again be due to differences in amount of sEVs loaded (Figure S11). Nevertheless, immuno-PCR sEVs from PE011 and PE020 showed IGF-1R amounts above LOD. With respect to HER2, a clear signal above LOD was monitored in sEVs from all four MPE samples, while Western blotting did not reveal any signal. Thus, the detection of EGFR was most robust, while HER2 also showed signals reliably above the methods LOD for all four sEV samples from MPE fluid of the advanced NSCLC patients. It is evident that sEVs from PE011 show a significantly higher relative receptor amount for all three targets than the other three sEV samples analyzed.

**Figure 5:**
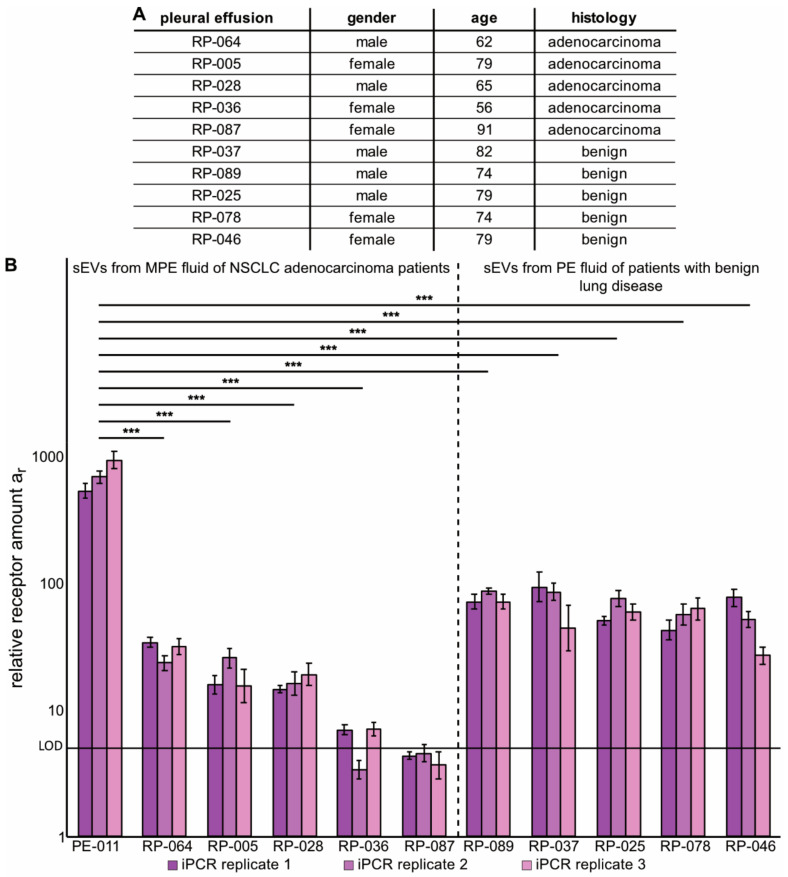
Immuno-PCR analysis of sEVs from MPE fluid of NSCLC patients and PE fluid from patients with benign disease. sEVs were isolated from PE fluid of five NSCLC adenocarcinoma patients and five patients with benign disease. (**A**): Summary of patient data matching the samples analyzed in B (additional data is presented in Table S4). (**B**): Relative amounts of EGFR on the retrospective patient-cohort in comparison to re-analyzed sEVs from the *EGFR*-mutated adenocarcinoma sample PE011 presented in Figure 4. Each bar represents the result of one immuno-PCR sample using 6 × 10^7^ sEVs isolated from patient samples, error bar = standard deviation of RT-PCR replicates, LOD = limit of detection. Significance levels are based on a one-way ANOVA test with subsequent Turkey multicomponent test, which was performed for the average of all three immuno-PCR results: ***p < 0.001 (all other sample pairs showed no significant difference, *p* > 0.05).

To further evaluate the capacity of the immuno-PCR towards EGFR detection, we analyzed a different set of PE-fluid samples (Figure 5). We selected five samples of PE fluid from patients with benign lung diseases and five samples of MPE fluid from NSCLC adenocarcinoma patients from a retrospective cohort of samples [39]. sEVs were isolated and characterized by NTA (Figure S12), as well as analyzed for CD9 expression to prove the presence of sEVs and lack of cellular contamination via calnexin by Western blotting (Figure S13), and equal numbers of sEVs were analyzed by immuno-PCR alongside new measurements of sEVs from PE011. Out of the five new MPE-fluid samples from NSCLC adenocarcinoma patients, EGFR could be detected above the method’s LOD in all but one sample. Again, PE011 showed a significantly higher signal for EGFR than all other samples, while there were no consistent differences in the EGFR expression level of sEVs between the analyzed MPE- and PE-fluid samples.

All in all, the presented data suggest that the developed immuno-PCR method based on affibody–DNA conjugates towards tumor-associated membrane receptors can be used for monitoring protein expression on sEVs in tumor liquid biopsies.

## 3. Discussion

With the recent development of precision cancer medicine there is great need for longitudinal monitoring of treatment response and selection of novel therapy approaches once refractoriness is evident. This is also true for NSCLC where TKIs targeting aberrant tumor-associated membrane receptors or fusion proteins, e.g., EGFR or ELM4-ALK, have reshaped the treatment landscape. Liquid biopsies of NSCLC tumors hold potential for treatment biomarker monitoring as they are noninvasive and can capture signaling from different metastatic sites [19,20]. In this study, we developed a diagnostic tool for sEV analyses to be used on liquid biopsies from NSCLC patients, an immuno-PCR based on affibody–DNA conjugates towards tumor-associated membrane receptors EGFR, HER2, and IGF-1R, all relevant as precision medicine targets in NSCLC and to some extent in other tumor types, e.g., breast cancer and/or as bypass drivers in TKI resistance development [11,14,17,18]. We show that this method can be used for monitoring protein alterations in sEVs from NSCLC cells and in MPE-fluid samples from NSCLC patients.

Central to the immuno-PCR developed in this work was the preparation of high-quality reagents. In many immuno-PCRs, antibody–DNA conjugates are used. However, the quality of antibodies may vary with regard to their sensitivity and specificity. Additionally, conjugation with DNA can modify the binding properties of antibodies, and commercial conjugation reactions often lead to a mixture of mono- and higher-order conjugates, which complicates not only the purification of antibody–DNA conjugates but also analyte quantification, as a single antibody-binding event can be detected through one or more DNA strands [27]. We therefore decided to use antibody-mimetics called affibodies. Affibodies are derived from a domain of staphylococcal protein A and selected towards their respective targets with various display techniques [26]. Although the development of affibodies is time consuming and early versions can have low affinity, the four affibodies used in this study are well characterized with regard to both affinity and selectivity (Table S1) and are thus a more promising starting point for high-quality DNA conjugates than some commercial antibodies [28,29,30,31,32,33,34]. In addition, the preparation of affibody–DNA conjugates was a straightforward process that yielded reagents of reproducible quality within a few days. The obtained conjugates with a defined 1:1 ratio and a labeling site outside of the target binding surface are in theory ideally suited for absolute quantification using a standard curve with matching DNA. However, we observed differences in amplification efficiencies for the affibody–DNA conjugates in the RT-PCR, which hampered absolute quantification. These different efficiencies may stem from intrinsic protein properties of the different affibodies, e.g., the affibody Z_EGFR_ used in the conjugate with the highest efficiency is negatively charged, whereas the other three affibodies carry a positive charge at neutral pH due to their different amino acid compositions. However, the presented analysis utilizing relative receptor amounts *a_r_* normalized to the background signal from each Z*_x_*–DNA conjugate allowed reliable detection of different expression levels of the same tumor-associated membrane receptor as shown in the analyses of sEVs from NSCLC cells treated with EGFR-targeting siRNA. This supports the idea that the method could be used for detecting overall changes of tumor-associated membrane receptors on sEVs during longitudinal treatment monitoring, although it has to be noted that the method does not reveal the heterogeneity of single sEVs with regards to receptor expression.

The lower limit of detection (LLOD) was determined to be in the range of 5 × 10^5^ NSCLC H1975-sEVs for EGFR, HER2, and IGF-1R, which is below commonly detected sEV amounts in plasma samples from cancer patients and therefore likely sufficient for analyses of sEVs with respect to tumor-associated membrane receptors from various cancers driven by these membrane receptors [36,40]. However, in this study, we tested the immuno-PCR method for these tumor-associated membrane receptors on sEVs from MPE fluid of NSCLC patients, as well as on sEVs from benign PE samples. MPE fluid is present in 10–15% of advanced NSCLC patients, and patients with adenocarcinoma subtype have a higher risk of MPE likely due to tumor localization in the periphery of the lung [37]. We and others have earlier shown that MPE fluid from NSCLC patients may be used as a source of biomarkers, e.g., ct-DNA or miRNA/mRNA in EVs [38,39]. Most often when MPE/PE fluid is taken to relieve the patient’s symptoms, at least 50 mL are obtained, and we only used 0.3–5 mL filtered MPE/PE fluid samples to isolate sEVs, showing that material is not a limiting factor for this type of analysis. We thereby demonstrated that the developed immuno-PCR method works for analyzing sEVs from MPE- and PE-fluid samples, and results indicate a possibility to detect expression differences of tumor-associated membrane receptor levels between samples. Even though methods with lower LLODs as well as single sEV analyses have been published, these methods are often better suited for the de novo detection of biomarkers, and little effort has been made to validate their reproducibility [24,25]. The interassay CV is a crucial number to assess the precision of an assay as it measures the variance of replicate samples in independent assay runs [41]. Especially when aiming for longitudinal treatment monitoring, a reasonably low interassay CV is needed to follow changes in the receptor amount on a given patient’s sEVs over time. The interassay CVs of the immuno-PCR were determined to be 14, 27, and 22% for Z_EGFR_–, Z_HER2_–, and Z_IGF-1R_–DNA, respectively, which we expect to be sufficient for reliably monitoring protein levels on sEVs from cancer patients. The presented immuno-PCR method already possesses the ability for medium throughput and multiplexing of samples as well as detection reagents, which can be further increased by the utilization of technical equipment. In case no affibody is available for an interesting sEV-based biomarker, the selection of further affibodies from combinatorial libraries or the use of antibody–DNA conjugates is possible.

In the field of NSCLC, the detection of cancer-driving oncogenes and acquired TKI-resistance during treatment is of high interest, but today, with respect to liquid biopsies, it is limited to ct-DNA with two FDA-approved platforms and to analyses of exosomal RNA [5,7,19,20]. The presented profiling of sEVs from MPE fluid of NSCLC patients with advanced disease, where targeted therapy is often used, revealed feasibility to monitor the tumor-associated membrane receptors EGFR, HER2, and IGF-1R. We observed higher EGFR expression levels in sEVs from PE011, which may reflect a true higher expression level of EGFR in these sEVs. However, it may also be a result of higher capturing of these sEVs to the magnetic beads, as this was based on CD9, which may differ among different clinical samples. The PE020 sEVs were also isolated from a patient with an *EGFR*-mutant NSCLC tumor, yet the immuno-PCR method detected much less signal for its EGFR content than in sEVs from PE011, which was consistent with Western blot profiling data in which PE011 had a higher EGFR level in sEVs relative to PE020. One may speculate this to be a result of a smaller fraction of EGFR-expressing tumor-derived sEVs in the liquid biopsy in PE020 than in PE011, which in turn may be a consequence of the different treatment lines among these two patients. We also evaluated EGFR expression levels on sEVs from PE samples from patients with benign lung disease in comparison to sEVs from MPE fluid from patients with NSCLC adenocarcinoma. Our results indicate that the EGFR expression level is clearly possible to evaluate in real clinical samples using our immuno-PCR method. Moreover, compared to samples from PE fluid of patients with benign lung disease, sEVs from PE011 clearly expressed a higher level of EGFR. With respect to MPE-fluid samples from the retrospective cohort of NSCLC adenocarcinoma patients, we did not find a higher level of EGFR as compared to PE-fluid samples from patients with benign lung disease. Our interpretation of this result is that these patients did not have EGFR-driven tumors and hence no elevated level of EGFR on sEVs isolated from the MPE fluid. Nevertheless, presented data support that the immuno-PCR method can be used for monitoring EGFR levels in sEVs and could potentially pick up alterations during treatment. However, we do not claim that the method is ready for early diagnostic or treatment monitoring purpose yet, as this needs to be validated in a larger liquid-biopsy sample cohort where the EGFR-TKI response is known and confirmed to be caused by alteration in EGFR levels and/or function.

We also observed a high signal of IGF-1R and HER2 in immuno-PCR of sEVs from PE011. This is interesting given that EGFR-TKI treatment has been shown to increase signaling via HER2 and IGF-1R in so-called bypass signaling implicated in EGFR-TKI resistance [4,11,17,18]. Moreover, it implicates that further evaluation of the immuno-PCR on sEVs from plasma of EGFR-TKI-treated patients upon relapse is warranted to advance this method towards clinical application. In summary, in the presented immuno-PCR method, different tumor-membrane receptors can be quantified on sEVs from liquid biopsies, e.g., MPE-fluid samples, which may offer a way to analyze possible bypassing signaling via detection of changes in the expression level of membrane receptors EGFR, HER2, and IGF-1R under TKI treatment. As *EGFR* and *ALK* mutations can be detected via genotyping of either ctDNA or exoRNA via RT-PCR or targeted sequencing methods [19,20,42,43], this could potentially be combined with the immuno-PCR method as its read-out step already is an RT-PCR.

## 4. Material and Methods

### 4.1. Cell Culture Conditions

The NSCLC cell line H1975 (ATCC^®^ CRL-5908™, LGC Standards, Teddington Middlesex, UK) of adenocarcinoma histology and harboring mutations in *EGFR (exon 20, T790M* and *exon 21, L858R)* was used in this study [44]. The cells were maintained in RPMI-1640 medium supplemented with fetal bovine serum (FBS, 10%) and 2 mM L-glutamine (Gibco, Life Technologies, Stockholm, Sweden). For isolation of sEVs from cell-culture media, cells were cultured in exosome-depleted FBS (#Gibco™ A2720801, Fisher Scientific, Gothenburg, Sweden) supplemented with 2 mM L-glutamine for 2 days. This was done as regular FBS may contain sEVs/exosomes that would blur results of sEVs secreted by the tumor cells.

### 4.2. EGFR siRNA Transfection

Endogenous EGFR expression in H1975 cells was reduced using transient siRNA transfection. Around 24 h post seeding when reaching 70% cell density, the cells were transfected with either 50 nM of EGFR siRNA (siRNA EGFR silencer siRNA Assay ID 42833, a mixture of 4 sequences) or 50 nM of Stealth RNAi™ (siRNA Negative Control, Med GC, 12935300), (both siRNAs were purchased from Fisher Scientific). The transfection was performed for about 4 h using a dilution of 1:50 in DharmaFECT 1 (Fisher Scientific) in serum-free RPMI media followed by a 20 h postincubation in FBS-containing media. Cells were thereafter cultured in exosome-depleted FBS (see Section 4.1) supplemented with 2 mM L-glutamine for 2 days. Afterwards, the media was collected for isolation of sEVs (see Section 4.3). Inhibition of EGFR expression in EGFR-siRNA treated cells was confirmed by Western blotting and flow cytometry analysis (see Section 4.7 and Section 4.8).

### 4.3. Isolation of sEVs from H1975 Cells

For sEV isolation from cell-culture media, approximately 50–75 mL was used. Media was centrifuged at 200 RCF for 5 min followed by centrifugation of the resulting supernatant at 720 RCF for 10 min. The samples were concentrated by Amicon Ultra-15 Centrifugal Filter Unit with a MWCO of 3 kDa (#UFC900324, Merck Chemicals and Life Science AB, Solna, Sweden) until reaching a volume of approximately 500 µL. sEV isolation was done by size-exclusion chromatography (SEC) on qEVoriginal columns (Izon Science, Oxford, UK) per manufacturer’s instruction. Briefly, columns were equilibrated with filtered PBS, and thereafter samples were gently added. Each sample was eluted in fractions of about 500 µL by adding PBS and fractions 6–10 were collected, pooled into a total volume of 2.5 mL, concentrated to 500 µL using Amicon^®^ Ultra-4 Centrifugal Filter Unit (MWCO = 3 kDa, #UFC800324, Merck Chemicals and Life Science AB), and subsequently subjected to nanoparticle tracking analysis (NTA) to verify particle size and amount. In an earlier study, sEV isolation by the same protocol was verified by scanning electron microscopy (SEM) showing the size and morphology of the isolated particles [45].

### 4.4. Collection and Isolation of sEVs from Malignant Pleural Effusion (MPE) Fluid of Advanced NSCLC Patients

For isolation of sEVs from MPE fluid, samples from four patients with NSCLC at Karolinska University Hospital, Stockholm, Sweden, were used. The study was run with ethical permit from The Ethics Review Authority in Sweden (https://etikprovningsmyndigheten.se, accesed on 8 March 2017), region Stockholm (EPN No. Dnr. 2016/2585-32/1, approval date 8th of March 2017), with patient informed consent to study participation and biobank approval.

All patients had NSCLC with adenocarcinoma histology and displayed different clinically validated genetic alterations: PE002: *EML4-ALK*, variant 3 (a/b), PE009: *KRAS*, exon 2, codon 12/13, PE011: *EGFR*, exon 21, *L858R* and PE020: *EGFR*, exon 20, *T790M*, exon 21, *L858R.* Further clinical characteristics are shown in Table S3. Different amounts of MPE fluid were obtained from each patient (PE002 ~800 mL, PE009 ~1500 mL, PE011 ~450 mL, and for PE020 ~450 mL). The MPE fluid was centrifuged at 2000 RCF for 5 min (Rotina38, Hettich Labinstrument AB, Stockholm, Sweden), and the supernatant was frozen at −80 °C and subsequently used for isolation of sEVs. The starting volume of MPE fluid for sEV isolation was ~5 mL, and the MPE fluid was first cleared by centrifugation (2000 RCF for 5 min), filtered (by 0.2 µm filter, Acrodisc^®^, Pall Corporations, VWR, Stockholm, Sweden), and concentrated to about 300–500 µL by Amicon^®^ Ultra-4 Centrifugal Filter Unit. The concentrated MPE fluid was then added to qEVoriginal columns and fraction 6–10 collected as described above. The sEV preparations were further concentrated prior to NTA analyses.

### 4.5. Isolation of sEVs from MPE Fluid Samples from NSCLC Adenocarcinoma Patients or from PE Fluid of Patients with Benign Lung Disease

A set of retrospective MPE/PE-fluid samples were selected from a second patient cohort [39]. Five MPE-fluid samples from NSCLC adenocarcinoma patients with different overall survival time and five PE-fluid samples from patients where no tumor cells were found in the fluid, hereafter referred to as benign, and were used (Table S4). The MPE or PE fluid was isolated in tubes without anticoagulating agents and within 1 h postsampling, centrifuged at 1500 RCF and +4 °C for 10 min. The supernatant was pipetted to a clean 14 mL Falcon tube and again centrifuged at 3000 RCF and +4 °C for 10 min to clear cells from the MPE/PE fluid, and the supernatants were stored at −80 °C until sEV isolation. For the isolation of sEVs from these samples ~0.7 to 1 mL, MPE- or PE fluid was used and filtered as indicated in Section 4.4. The resulting material of ~0.3 to 0.5 mL was loaded on the qEVoriginal columns and further processed as indicated in Section 4.4.

### 4.6. Analyses of sEV Size and Amount by Nanoparticle Tracking Analysis (NTA)

The sEV size and amount were analyzed by NTA on a NanoSight NS300 (Malvern Panalytical, Malvern, UK). The sEV samples were diluted in filtered PBS and analyzed with the following settings: sample dilution 1:20 to 1:50, syringe pump speed: 100 (all samples), and camera level: 13–15 detection threshold for analysis set at 5 or 8 with time for analysis 3 × 60 s (all samples).

### 4.7. Western Blot Analyses of sEVs

Protein extraction from H1975 or MPE-derived tumor cells was done using 1x RIPA buffer (50 mM Tris-HCl pH 7.4, 150 mM NaCl, 1% Triton-X100, 1 mM EDTA, 0.1% SDS) supplemented with protease and phosphatase inhibitors (complete Mini; Phos STOP, Sigma Aldrich, Merck Chemicals and Life Science AB). Samples solubilized in RIPA buffer were incubated on ice (about 15 min) and cleared form cell debris by centrifugation (Micro 200R, Hettich Labinstrument AB) at 24,100 RCF. Protein concentration was assessed by BCA assay (Pierce™ BCA Protein Assay, Fisher Scientific), and 50 µg were used for Western blot profiling. For protein extraction of sEVs, 5x RIPA was used, giving a final concentration of 1x RIPA, and 12–25 µl were used for each Western blot. Thus, the total amount of sEVs analyzed ranged from about 9 × 10^7^ to about 7 × 10^8^. Proteins were loaded on Invitrogen NuPAGE^®^ gel system (Fisher Scientific) by mixing the RIPA-samples with sample buffer and reducing agent followed by heating (10 min, 70 °C). Proteins were separated (1h, 180V) on 4–12% Bis-Tris (TB) gels with MES as running buffer or on 3–8% Tris-Acetate (TA) gels with Tris-Acetate SDS running buffer (all reagents from NuPAGE^®^, Fisher Scientific). Proteins were blotted onto Odyssey^®^ Nitrocellulose Membrane (LI-COR GmbH, Bad Homburg, Germany) in transfer buffer with 10% methanol (30V, 90 min). Membranes were blocked in Odyssey^®^ blocking buffer (LI-COR GmbH)/TBS-T 1:1 for 1 h and probed with primary antibodies (overnight at 4 °C) (full details found in Table S5): anti-CD9 (CD9 (D3H4P) Rabbit mAb#13403), anti-calnexin (Calnexin antibody, #2433), anti-EGFR (EGF Receptor (D38B1) XP^®^Rabbit mAb; #4267), and anti-IGF-I Receptor β (IGF-I Receptor β Antibody, #3027) (all from Cell Signaling Techology, BioNordica AB, Stockholm, Sweden) as well as anti-TSG101 (Recombinant-Anti-TSG101 antibody[EPR7130(B)], Ab125011, Abcam, Cambridge, UK). Anti-β-tubulin I (Monoclonal Anti-β-Tubulin antibody, #T7816, Sigma-Aldrich, Merck Chemicals and Life Science AB) was used as loading control for the cell samples. After washing in TBS-T buffer (about 3 × 5 min), membranes were probed with goat anti-Rabbit IRDye^®^ 800CW antibody (IRDye^®^ 800CW goat anti-Rabbit IgG Secondary Antibody, #926-32211), or goat anti-mouse IRDye^®^ 680 (IRDye^®^ 680RD Donkey anti-Mouse IgG Secondary Antibody, #926-68072); both from LI-COR GmbH). After washing in TBS-T buffer for about 3 × 5 min resulting signals were visualized as well as quantified on the Odyssey^®^ Sa Infrared Imaging System (LI-COR GmbH).

### 4.8. FACS Assessment of EGFR Expression After siRNA Treatment

For evaluation of EGFR expression in H1975 cells prior to and post EGFR siRNA, transfection, cells were fixed after harvest in 4% paraformaldehyde solution in PBS for approximately 10 min at room temperature. Cells were thereafter rinsed in PBS and resuspended in PBS containing digitonin to a final concentration of 100 µg/mL. For staining of EGFR, an anti-EGFR antibody (EGF Receptor (D38B1) XP^®^ Rabbit mAb #4267 (Cell Signaling Technology) generated by immunizing animals with a fusion protein containing the cytoplasmic domain of human EGFR was used at dilution 1:100. Staining was performed overnight at 4 °C, and samples were thereafter washed and incubated with the secondary antibody conjugated to Alexa Fluor 488 (Goat anti-Rabbit IgG (H+L) Cross-Adsorbed Secondary Antibody, # Invitrogen A11008, Fisher Scientific AB) at a dilution 1:1500 for 30 min in the dark at room temperature.

The specificity of this staining procedure was established in untreated H1975 cells using the same staining procedure as above but including unstained cells and an isotype control (rabbit (DA1E) mAb IgG XP^®^ Isotype Control #3900; Cell Signaling Technology) in a dilution of 1:100 followed by secondary antibody as indicated above. Unstained cells differed from cells stained with either secondary antibody only or isotype antibody followed by secondary antibody, which in turn did not clearly differ from each other [46]. However, all three of these conditions showed a clear difference in comparison to cells stained with the primary EGFR antibody followed by secondary antibody. Thus, for the presented data in Figure S7, cells stained only with the secondary antibody were compared to those where the EGFR antibody was applied followed by the secondary antibody. The samples were analyzed on a NovoCyte 3000 Flow cytometer (Acea Bioscience/Agilent, Santa Clara, CA, USA). The signal from Alexa 488 was measured, and the histogram and mean intensity unit was used for data interpretation.

### 4.9. Production of Z_x_–DNA-Conjugates

For detection of transmembrane receptors EGFR, HER2, HER3, and IGF-1R, the affibodies Z_02377_, Z_Her2:342_, Z_08698_ and Z_4551_ were used [31,32,33,34], which are named Z_EGFR_, Z_HER2_, Z_HER3_ and Z_IGF-1R_, respectively, throughout this manuscript. Affibody sequences were cloned into a pET21a vector to carry a C-terminal Sortase A recognition motif (a.a. sequence: LPETGG) directly followed by a H_6_-Tag. Sequence-confirmed vectors were transformed into *E. coli* BL21 DE3 cells, which were used to inoculate 40 mL TBS-Y cultures with 100 µg/mL ampicillin. Cultures were grown at 37 °C and induced by addition of isopropyl-β-d-thiogalactopyranoside (IPTG) to 1 mM after reaching an OD_600_ of 0.8–1.0. Expression was performed at 37 °C for 18 h before harvest via centrifugation (4000 rcf, 10 min) and resuspension of pellets in 2.5 mL binding buffer (300 mM NaCl, 10 mM imidazole, 50 mM Na_2_HPO_4_, pH 7.4). Cell suspensions were lysed at 95 °C for 10 min and cleared via centrifugation (12000 rcf, 4 °C, 30 min). The supernatant was added to 0.25 mL columns of HisPur Cobalt Resin (89966, Fisher Scientific) equilibrated with binding buffer. After washing with 6 column volumes (CVs) binding buffer, affibodies were eluted using 1 mL elution buffer (300 mM NaCl, 300 mM imidazole, 50 mM Na_2_HPO_4_, pH 7.4) and lyophilized. Protein pellets were dissolved in pure MilliQ water and buffer exchanged to Sortase A buffer (150 mM NaCl, 10 mM CaCl_2_, 50 mM Tris, pH 7.5) using PD-10 columns (17-0851-01, VWR) according to manufacturer’s instructions. Protein concentrations were determined via 2-D Quant Kit (80-6483-56, GE Healthcare, Freiburg, Germany), and the integrity of proteins was determined using MALDI-TOF measurements on a 4800 MALDI TOF/TOF Analyzer (Sciex, Framingham, MA, USA). Proteins were coupled to the 5′-G_3_-modified desoxyoligonucleotide CTA ACA GGA TTC AGG TAA TAG CCC GGT TTT AGA ACA CGA CAA GAT in a 300 µL scale as previously published [27]. Briefly, 7.5 nmol of each affibody were mixed with 3 nmol DNA in 300 µL Sortase A buffer with 300 µM Ni(II)acetate and incubated for 30 min at 37 °C after addition of 1 pmol of Sortase A3* (P94S, D160N, K196T). Z*_x_*–DNA conjugates were purified via HPLC on a Zorbax 300SB-C18 column (3.5 μm, 4.6 × 150 mm, Agilent, Stockholm, Sweden) with a gradient of 30–50% solvent B (acetonitrile) in solvent A (25 mM triethylammonium, pH 7.5) for Z_EGFR_–DNA and 20–40% for Z_HER2/HER3/IGF-1R_–DNA. Conjugate fractions were pooled into low protein binding tubes (90410, Fisher Scientific), lyophilized, solved in PBS-T (137 mM NaCl, 2.7 mM KCl, 10 mM Na_2_HPO_4_, 0.2 mM K_2_HPO_4_, 0.1% Tween-20 pH 7.4) to 1 µM (based on absorbance at 260 nm and the molar extinction coefficient for the incorporated DNA sequence, $\varepsilon_{260}=522900\;\frac{L}{mol\;\times \;cm}$), and aliquoted in portions of 6 µL before being stored at –80 °C. The purity of all Z*_x_*–DNA conjugates was confirmed via analytical HPLC.

### 4.10. Measurement of Amplification Efficiencies for Z_x_–DNAs via RT-PCR

A fresh aliquot of each Z*_x_*–DNA conjugate as well as unmodified template DNA stored at −80 °C was diluted 1:100 into PBS-T followed by a 10-fold dilution series in nuclease-free distilled water (11538646, Fisher Scientific) to reach working concentrations of 1 pM to 1 fM. 26.4 µL of each working solution were then added to 39.6 µL RT-PCR mastermix, which contained 500 nM of each RT-PCR primer (sequences CTA ACA GGA TTC AGG TA and ATC TTG TCG TGT TCT AA for forward and reversed primer, respectively) in iQ SYBR Green Supermix (1708882, Bio-Rad, Solna, Sweden). Each RT-PCR sample was divided in three 20 µL replicates on a RT-PCR plate (HSP9601, Bio-Rad), and analysis was performed on a CFX96 Real-Time PCR detection system (Bio-Rad) using an initial denaturation of 2 min at 95 °C followed by 40 cycles of 10 s at 95 °C, 15 s at 51 °C, and 30 s at 72 °C. Fluorescence was measured only in the last step of each cycle. A nontemplate control (NTC) was included.

### 4.11. Detection of sEVs via Immuno-PCR

Immuno-PCR was performed on sEVs isolated from NSCLC H1975 cell-culture medium or MPEs from NSCLC patients or from PE fluid of patients with benign lung disease (see above). Isolated sEVs were obtained in 1x PBS and diluted into PBS-C (1x PBS with 0.05% casein (*w*/*v*)) to defined particle counts. For the dilution series, concentrations of 2 × 10^6^, 1 × 10^6^, 4 × 10^5^, 2 × 10^5^, 1 × 10^5^, 4 × 10^4^, 2 × 10^4^ and 1 × 10^4^ particles/µL were used, respectively. For analysis of the effect of siRNA treatment on H1975 cells and for analysis of MPE- or PE-fluid samples, sEVs were adjusted to a concentration of 1 × 10^6^ and 4 × 10^6^ particles/µL, respectively.

After sEV sample preparation, immuno-PCRs consisted of (1) capture of sEVs on anti-CD9 magnetic beads from the exosome-human CD9 isolation reagent (15450544, Fisher Scientific), (2) incubation of bead-captured sEVs with affibody–DNA conjugates, (3) thorough washing and resuspension of the beads into nuclease-free water, and (4) analysis of bead suspensions via RT-PCR. All incubation steps were performed in screw-cap vials (72.693.100, Sarstedt, Nürnbrecht, Germany) or 96-well plates (391-3615, VWR), which were blocked overnight with PBS-C. For capturing 6 µL of the commercial anti-CD9 bead suspension were added to one vial with 1 mL PBS-C. Subsequent stated volumes refer to 6 µL of initial bead suspension but were usually scaled up to probe the same sEV sample with different Z*_x_*–DNAs in parallel. To measure background binding of different Z*_x_*–DNAs, a bead sample without sEVs was analyzed in parallel. The PBS-C was removed, and beads were mixed with 15 µL of sEV sample before incubation at 4 °C for 18 h while rotating. Beads were washed with 1 mL of PBS-C before being suspended in 75 µL of PBS-C and transferred in a 96-well plate prefilled with 125 µL PBS-C per well. A working solution of 1 nM affibody–DNA conjugate in PBS-CT (1x PBS with 0.05% casein (*w*/*v*) and 0.1% Tween-20 (*v*/*v*)) was prepared by diluting a freshly thawed 1 µM aliquot 1:100 into PBS-T and then 1:10 into PBS-CT. All buffer was removed from the beads, and 10 µL of affibody–DNA conjugate solution were added and incubated at room temperature for 1 h while gently shaking. Afterwards 125 µL of PBS-CT was added, the beads were collected on a magnet for one minute, and directly afterwards, all supernatants were removed. Addition of 125 µL PBS-CT and one minute of magnetic bead capture was repeated before the beads were resuspended in 50 µL PBS-CT and directly transferred to a new well on the 96-well plate prefilled with 125 µL PBS-CT. After bead capturing for one minute the supernatant was again removed, and bead washing with 125 µL PBS-CT and one minute of magnetic capture were repeated before transferring the beads again using 50 µL PBS-CT for resuspension. After another washing step with 125 µL PBS-CT and a magnetic capturing step for one minute, all supernatants were removed, and 26.4 µL of nuclease-free water was added. The beads were resuspended and added to 39.6 µL RT-PCR mastermix with 500 nM of each RT-PCR primer in iQ SYBR Green Supermix, divided in three 20 µL replicates on a RT-PCR plate, and measured as described above. In all immuno-PCR runs a standard curve of nonconjugated DNA-template in a 10-fold dilution from 0.1 nM to 0.1 fM, as well as a nontemplate control (NTC) were included.

### 4.12. Data Analysis

Raw data of RT-PCR runs was analyzed with the CFX manager software (vs. 3.1, Bio-Rad) using a threshold of 60. Only runs in which the NTC showed a Cq of at least two cycles lower than the lowest nonconjugated DNA standard were used. Analysis of amplification efficiencies for Z–DNAs was performed with the automatic calculation of efficiency and precision for standard samples with the CFX manager software. In immuno-PCR runs only measurements for which the Cq lay in the linear range of the standard curve (10 pM to 1 fM of template DNA) were used, and for cell-line-derived sEV samples only measurements for which the standard deviation (SD) was below 0.2 Cq values were further analyzed. In cases were a single replicate of the RT-PCR was a clear outlier, only the two other replicates were used. All downstream calculations were based on the average Cq values for RT-PCR replicates and their standard deviation as calculated by the CFX manager software. To obtain relative amounts *a_r_* the ΔCq was calculated as

∆Cq = Cq (beads with sEVs)−Cq (beads without sEVs)(1)

with

SD (∆Cq) = SD (Cq (beads with sEV)) + SD (Cq (beads without sEVs))(2)

and transferred to a linear scale via

a_r_ = 2^-∆Cq^ (3)

with

SD(a_r_) = 2^−∆Cq ± SD(∆Cq)^ (4)

The methods limit of detection (LOD) for relative amounts *a_r_* was calculated as

LOD = background signal + 3∙SD (background signal)(5)

which transfers to

LOD = 2^[Cq(beads without sEVs) − Cq(beads without sEVs)]^ + 3 × 2^(SD(Cq)+SD(Cq))^ (6)

The SD(Cq) was set to 0.2, as this was the threshold SD for excluding RT-PCR measurements, which simplifies the calculation to

LOD = 1+3 [2^(0.4)^] = 4.96 (7)

The lower limit of detection (LLOD) for the sEV-amount was calculated for all four affibody–DNA conjugates by plotting the mean value of the mean *a_r_* values for all immuno-PCR replicates shown in the dilution series against the amount of NSCLC H1975 cell sEVs. A linear regression analysis was performed in Microsoft Excel (vs. 16.11, Microsoft, Albuquerque, NM, USA), and the LLOD was calculated as the intersection of the regression line with the method LOD. The interassay CV was calculated by first determining the mean of the mean *a_r_* values for all replicates above the LOD, as shown in the dilution series, as well as their standard deviation SD (means), and then calculating

CV_inter_ = SD(means)/means (8)

Statistical analysis for determination of significance levels was performed to compare samples with detection of the same membrane receptor using the one-way ANOVA test with subsequent Turkey multicomponent test from GraphPad Prism (vs. 5.03, GraphPad Software Inc., San Diego, CA, USA). As input, the relative receptor amount *a_r_* as well as its higher and lower limits

limits(a_r_) = a_r_ ± SD(a_r_) (9)

were used.

## 5. Conclusions

In this study, we developed a method to detect sEVs based on immuno-PCR analyses utilizing affibody–DNA conjugates towards the tumor-associated surface receptors EGFR, HER2, and IGF-1R. We applied this method for analyses of sEVs isolated from the NSCLC cell line H1975 as well as from MPE fluid from NSCLC adenocarcinoma patients and PE fluid from patients with benign disease. The lower limits of detection (LLOD) for all three markers were determined to be well below the expected number of sEVs in patient liquid biopsy samples, e.g., plasma [36]. Our analyses of MPE- or PE-fluid samples also show the ability to detect these tumor-associated membrane receptors on sEVs in sample volumes that are feasible to obtain in a clinical setting. Moreover, the interassay CVs of the developed immuno-PCR are expected to be sufficiently low to allow assessment of alteration in tumor-associated membrane receptors on sEVs from NSCLC liquid biopsies. This indicates possible future development of affibody–DNA-based immuno-PCRs as a tool for longitudinal treatment monitoring of sEVs from advanced NSCLC patients, for example. using either plasma or MPE fluid as a source of sEVs.

## Data Availability

The data generated during the current study are available from the corresponding author upon reasonable request.

## Supplementary Materials

The following are available online at https://www.mdpi.com/2072-6694/13/4/922/s1, Figure S1: Matrix-assisted laser desorption/ionization time-of-flight (MALDI-TOF) analysis of affibodies purified via immobilized metal affinity chromatography (IMAC) after *E. coli* expression. Figure S2: Analytical reversed-phase high performance liquid chromatography (RP-HPLC) of purified affibody–DNA conjugates. Figure S3: Western blot analysis of sEVs derived from cell culture media of H1975 cells. Figure S4: Nanoparticle tracking analysis (NTA) of cell-line-derived sEVs. Figure S5: Immuno-PCR analysis of HER3 on three dilution series of sEVs isolated from conditioned cell culture medium of H1975 cells with ZHER3–DNA. Figure S6: Regression analysis of the dilution series of sEVs from H1975 conditioned cell-culture media to determine the LLOD of each Zx–DNA conjugate. Figure S7: Flow cytometry analysis of EGFR expression in H1975 cells. Figure S8: Western blot analysis of H1975 cells and corresponding sEVs from conditioned cell-culture media after EGFR-siRNA treatment. Figure S9: Full analysis of the biological replicates of sEVs from the NSCLC H1975 conditioned cell-culture media after siRNA treatment of cells. H1975 cells were treated with either EGFR-targeting or non-targeting siRNA, and sEVs were isolated from conditioned cell-culture media. Figure S10: Nanoparticle tracking analysis (NTA) of sEVs isolated from MPE fluid of advanced NSCLC patients. Figure S11: Western blot analysis of sEVs derived from MPE fluid of advanced NSCLC patients and corresponding tumor cells. Figure S12: Nanoparticle tracking analysis (NTA) of sEVs isolated from MPE fluid of NSCLC patients and from PE fluid of patients with benign disease. Figure S13: Western blot analysis of sEVs derived from MPE fluid of NSCLC patients or PE fluid from patients with benign disease. Table S1: Summary of properties for affibody proteins used in this study. Table S2: Summary of the efficiencies of different Zx–DNA conjugates used in the immuno-PCR assay. Table S3: Summary of the clinical and molecular features of advanced NSCLC patients. Table S4: Summary of the clinical characterization of the retrospective cohort of patients from which MPE and PE fluid was obtained as source of sEVs. Table S5: Information of primary- and secondary antibodies used in Western blot analyses.
